# Supplementary material for: NF-κB–Dependent Snail Expression Promotes Epithelial–Mesenchymal Transition in Mastitis
Source: Animals (Basel). 2021 Dec 1;11(12):3422. doi: 10.3390/ani11123422 (PMC8698035; doi:10.3390/ani11123422)
Supplement: Supplementary file 1 [file animals-11-03422-s001.zip › animals-1439215-supplementary materials-authors.pdf]

E-cadherin

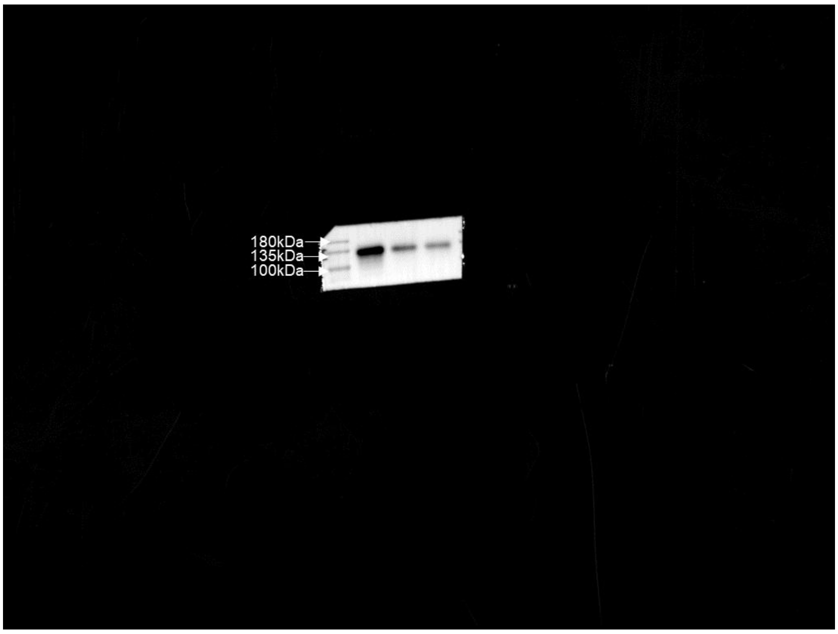

N-cadherin

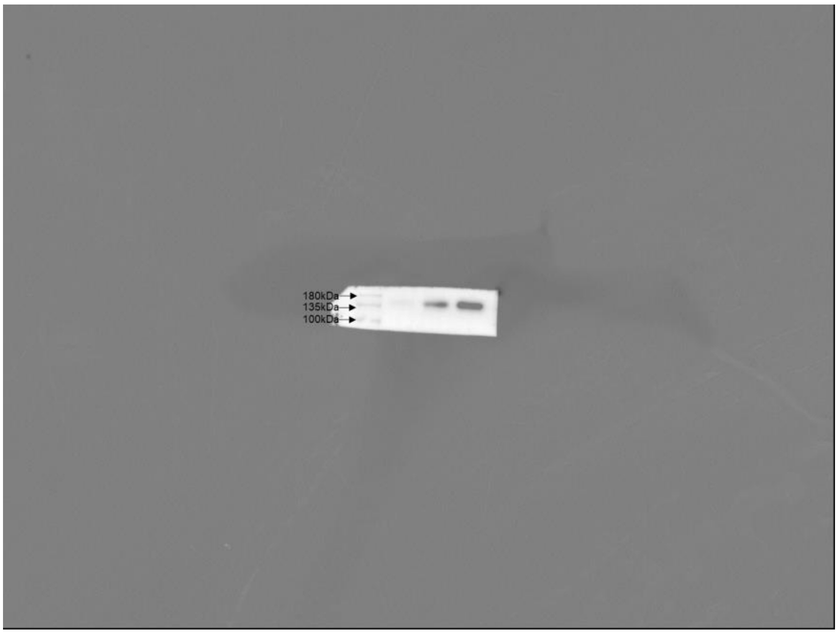

Vimentin

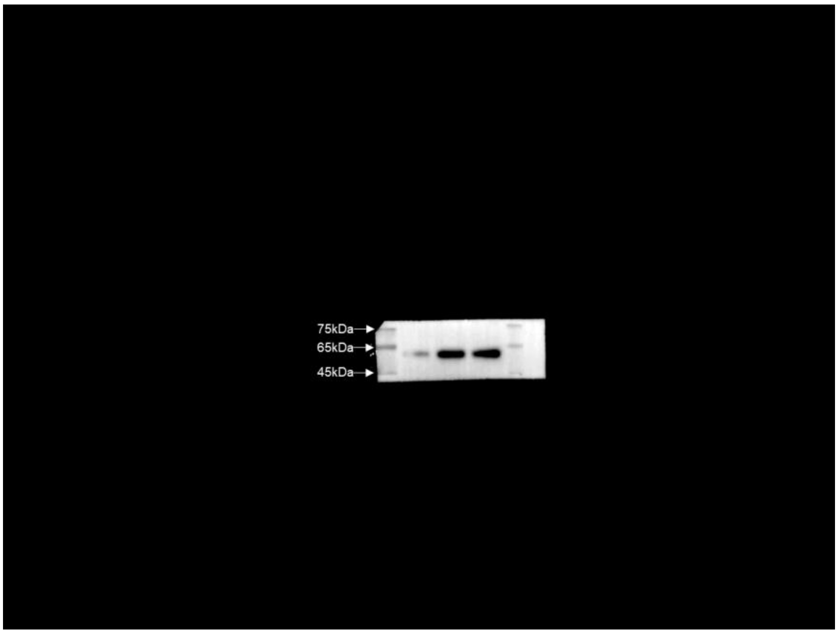

Collagen Ⅲ

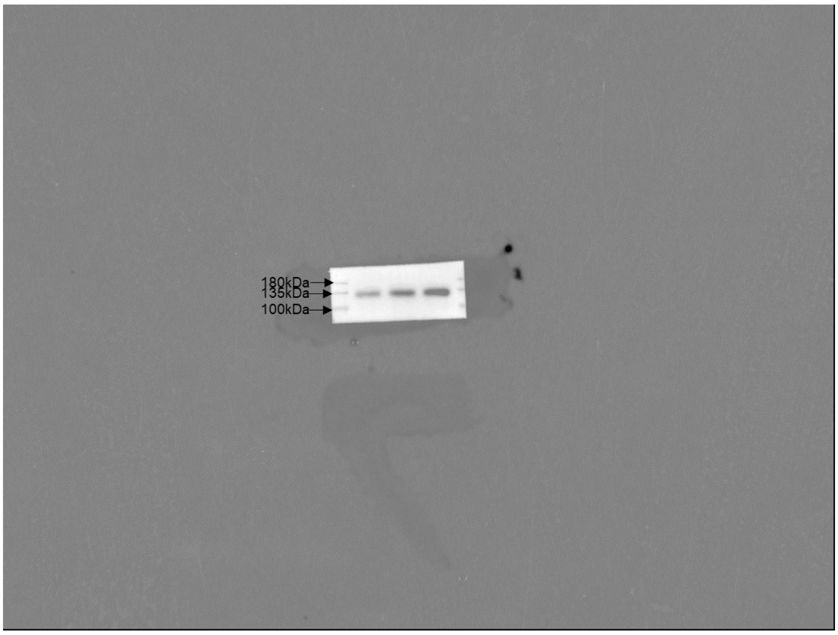

GAPDH

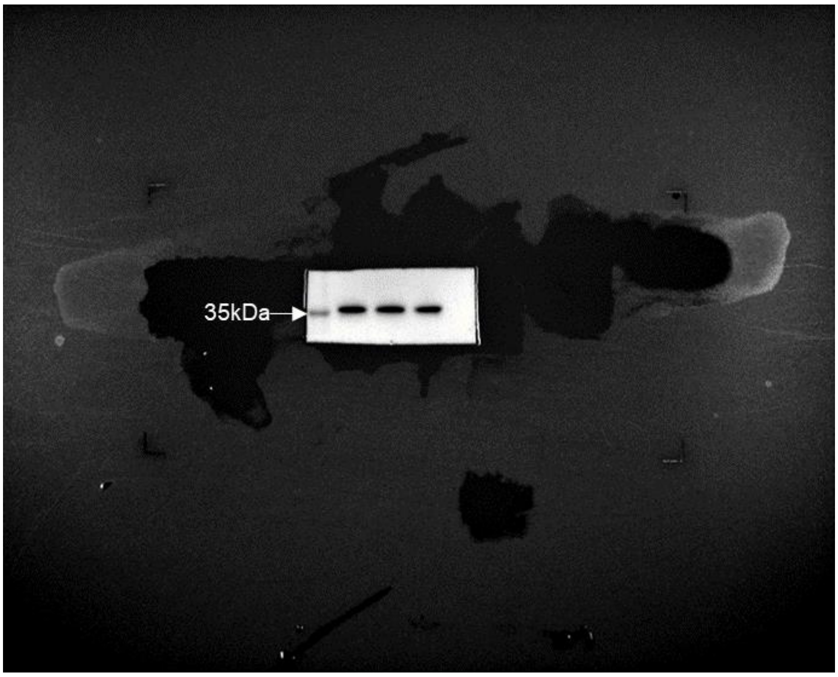

Figure S1 Original western blot figures for figure 1E.

E-cadherin

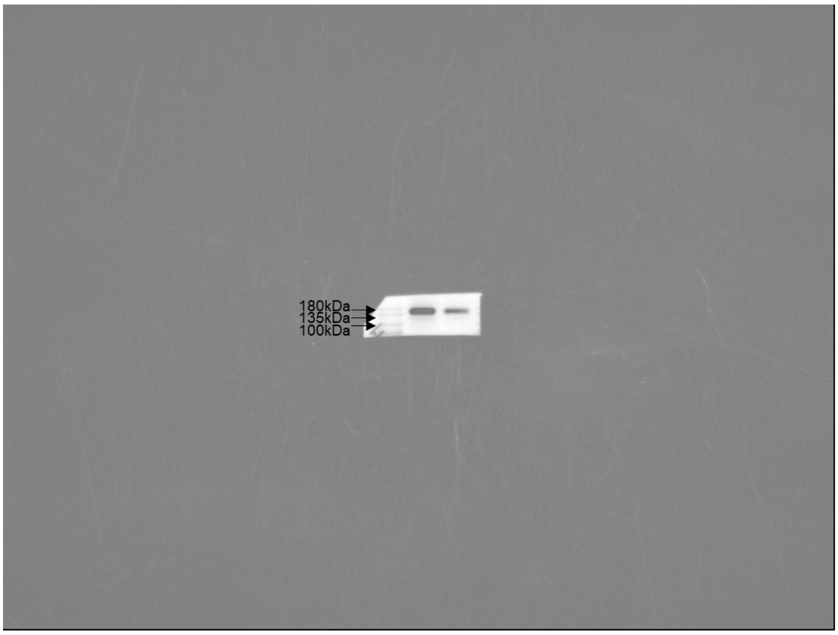

pp65

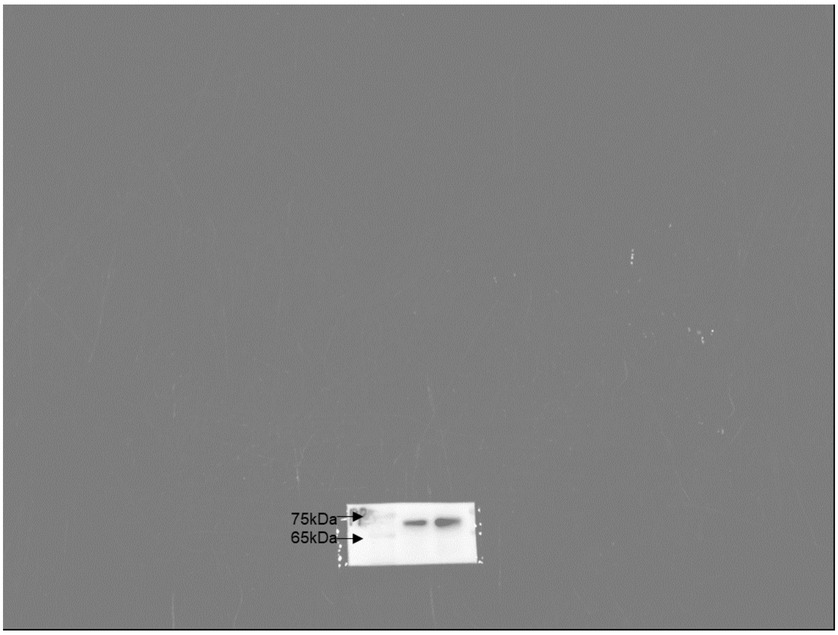

p65

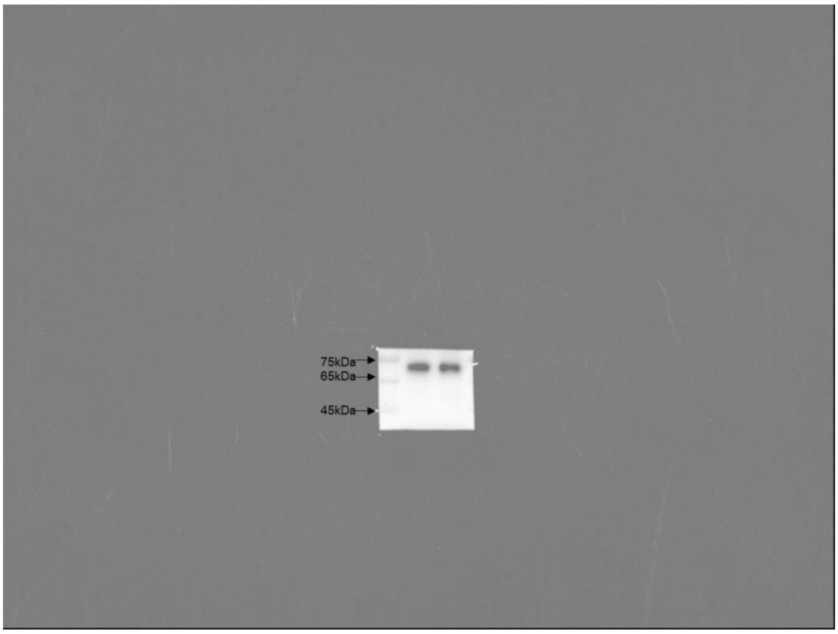

Snail

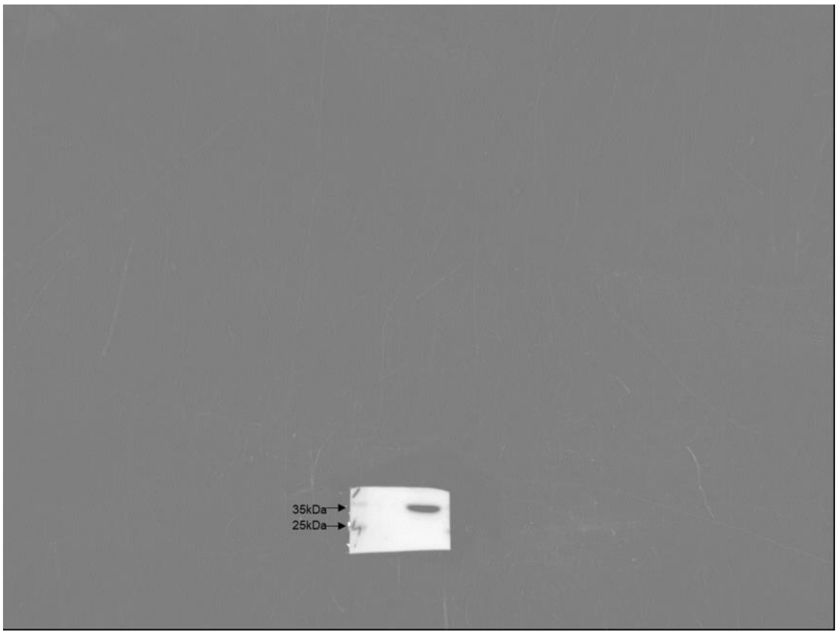

GAPDH

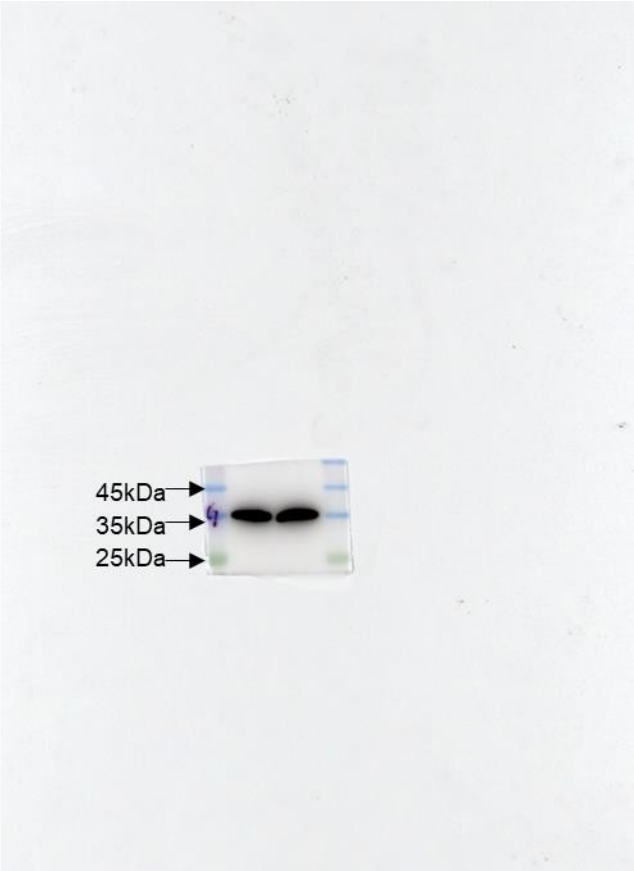

Figure S2 Original western blot figures for figure 2A.

pp65

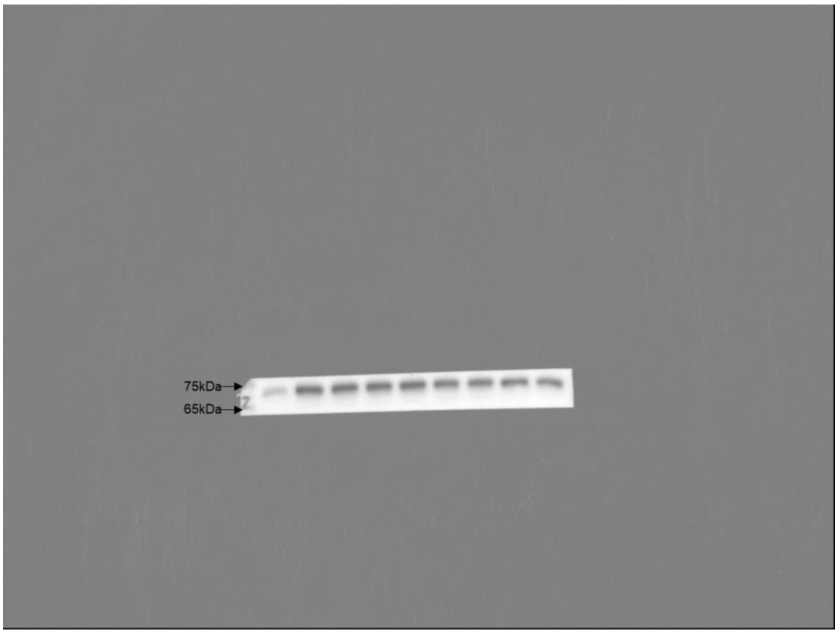

p65

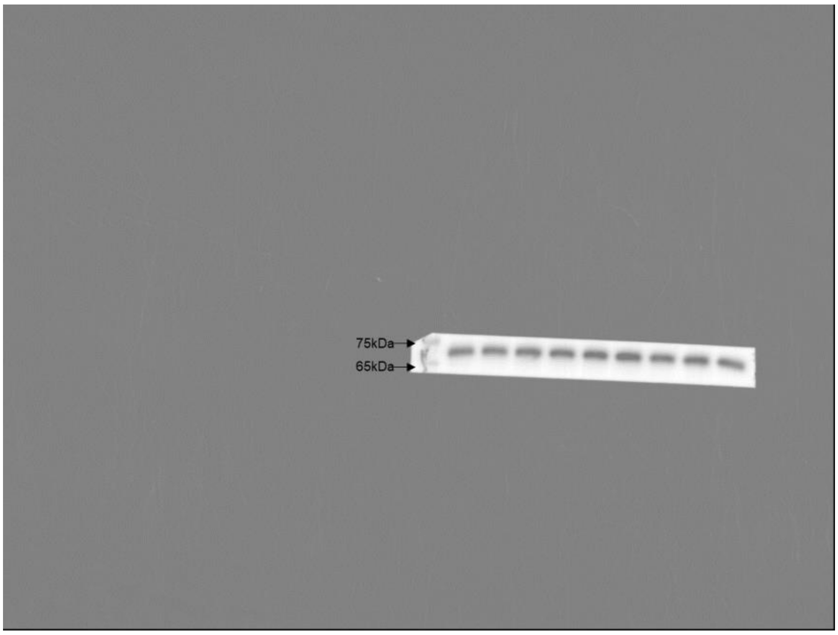

Snail

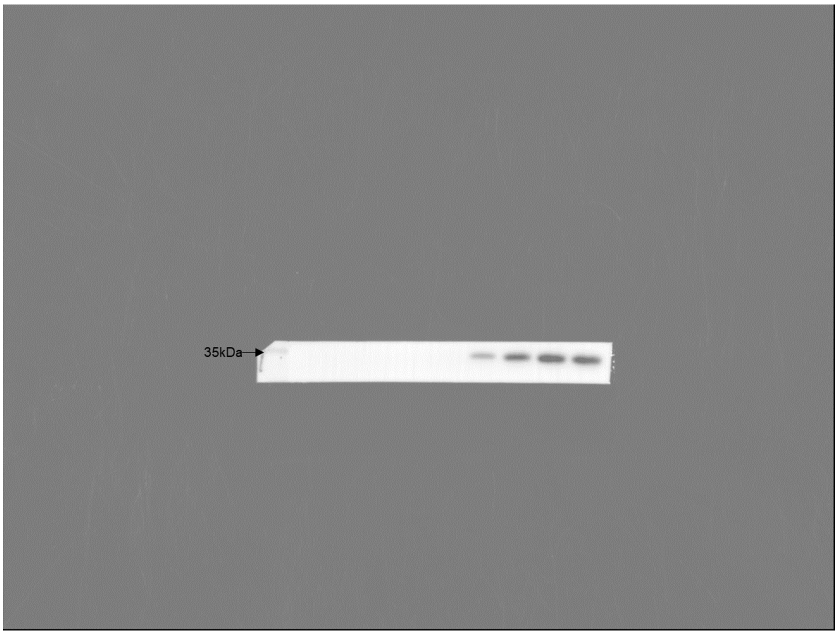

GAPDH

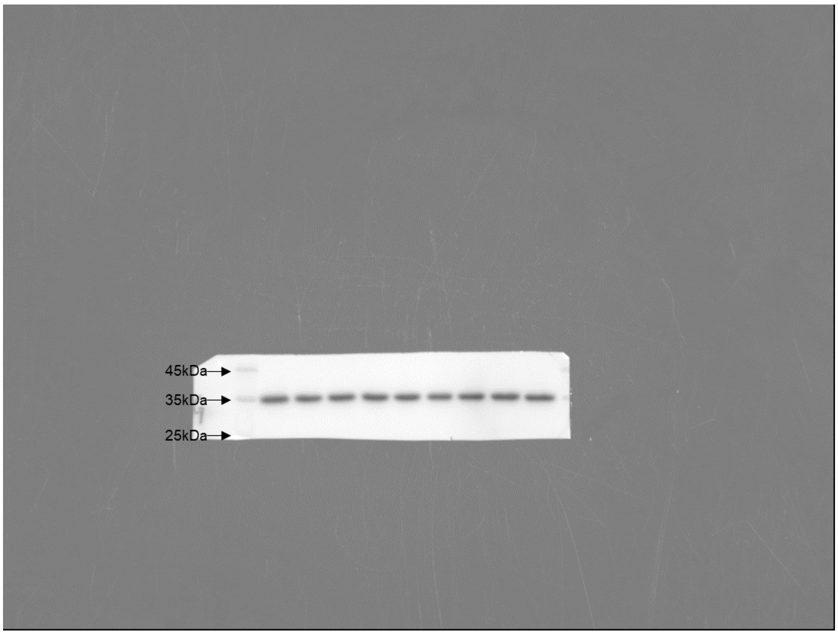

Figure S3 Original western blot figures for figure 2C.

pp65

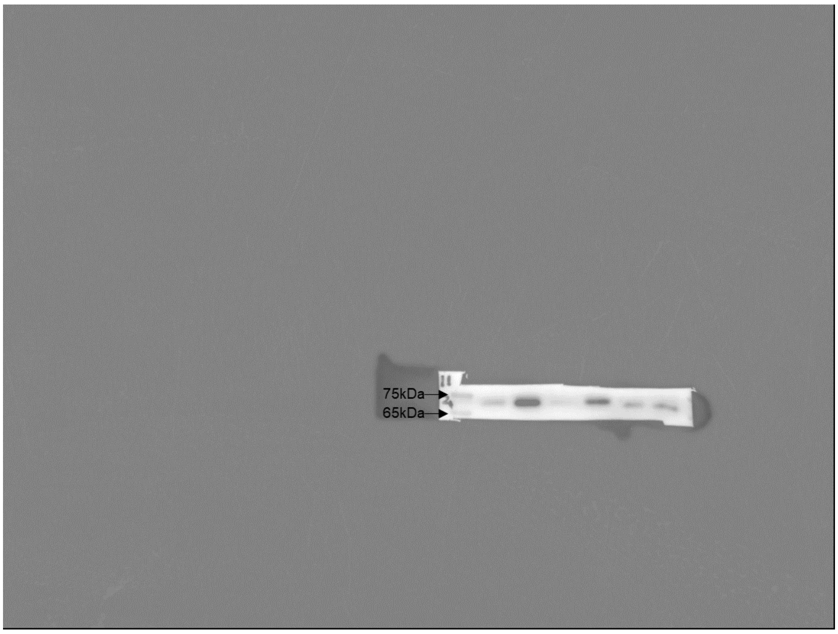

p65

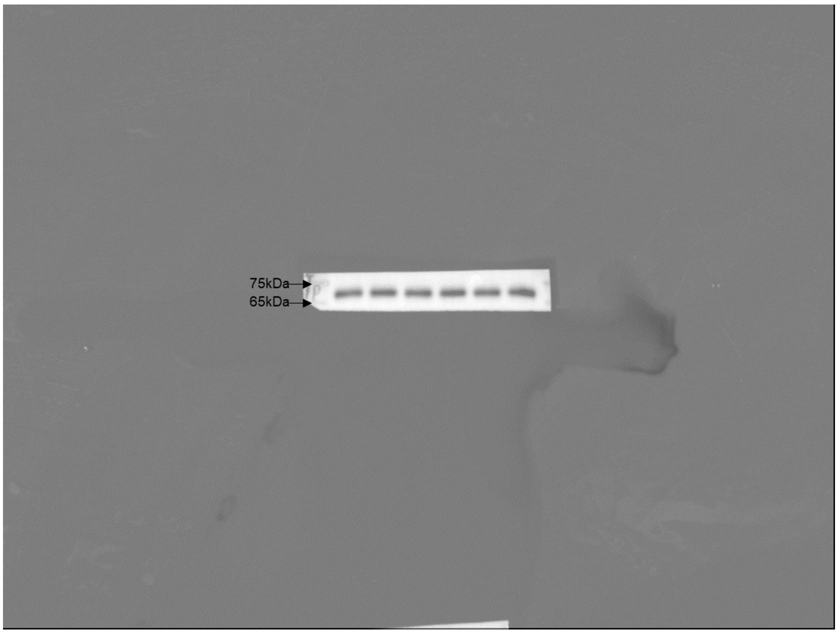

Snail

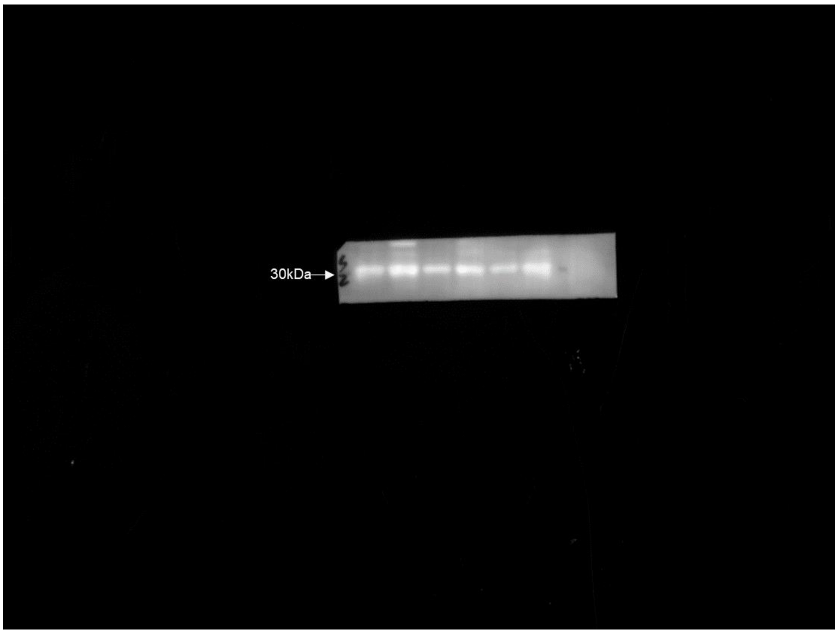

GAPDH

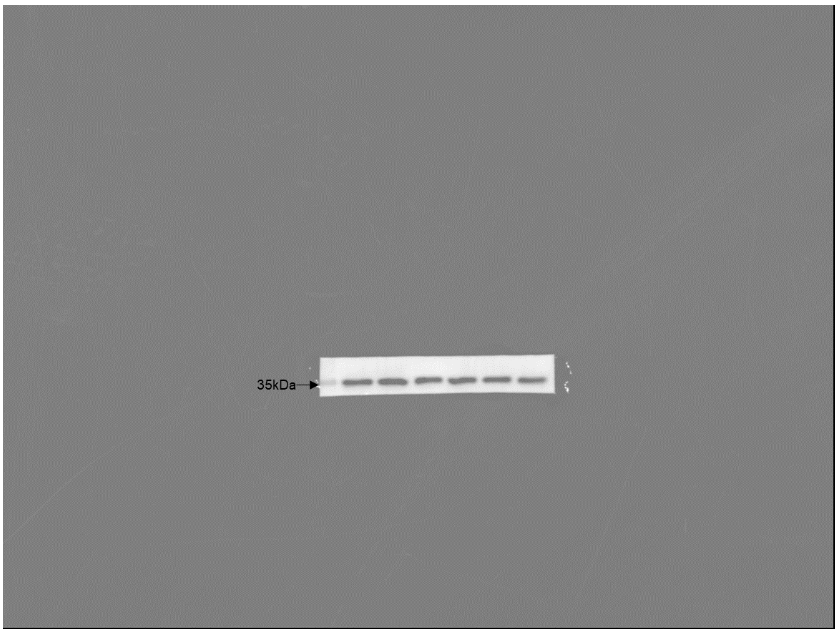

Figure S4 Original western blot figures for figure 2D.

pp65 - total protein

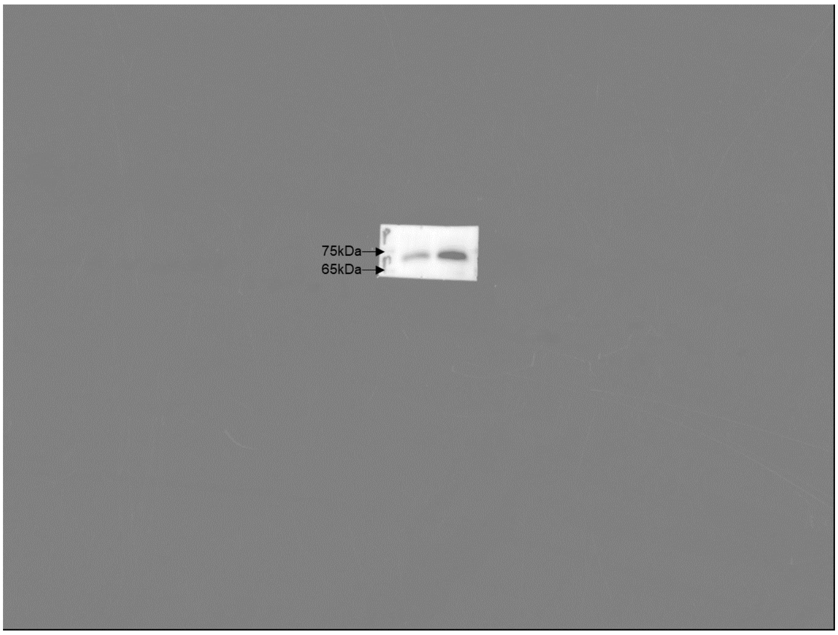

p65 - total protein

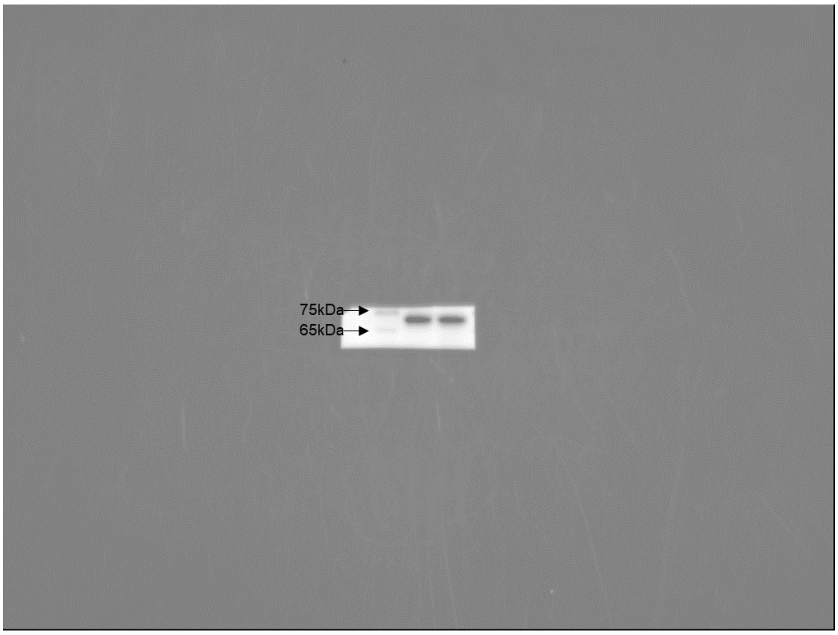

Snail- total protein

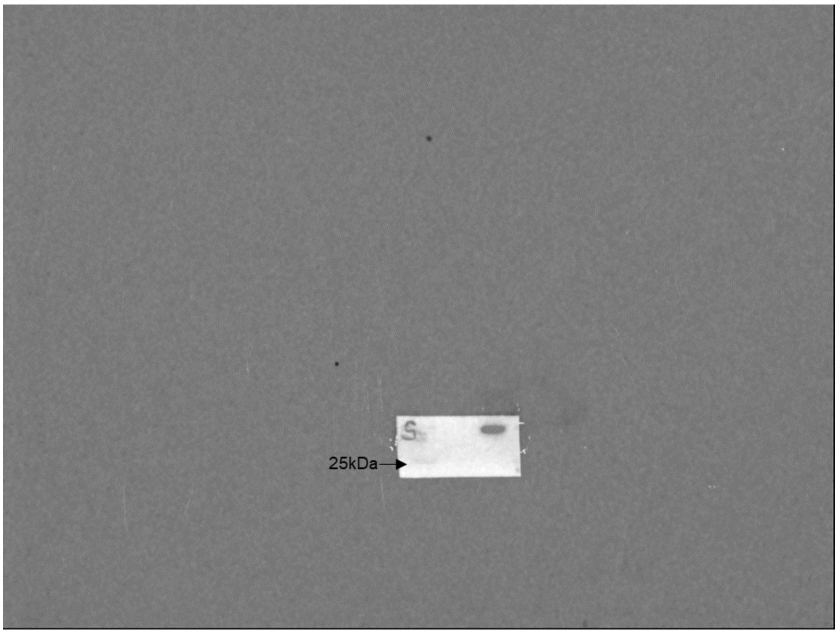

GAPDH - total protein

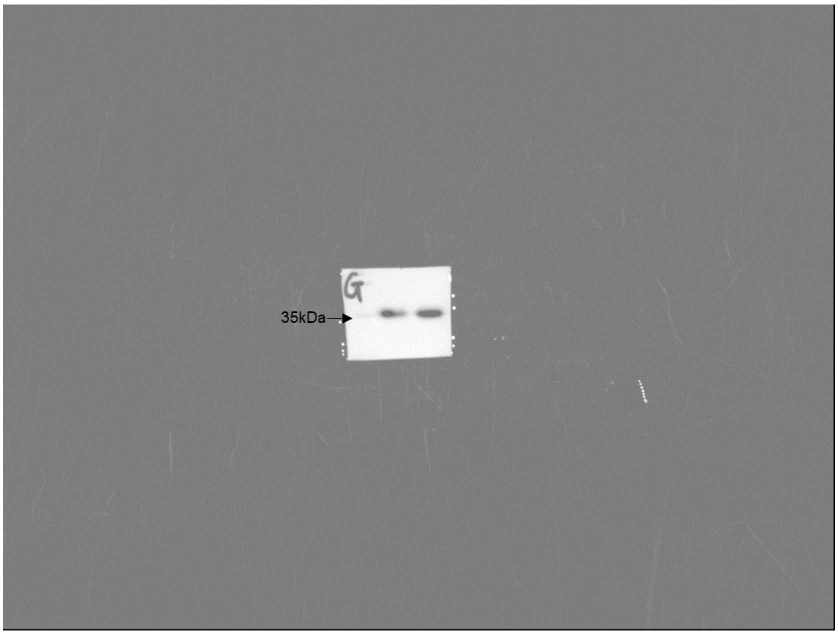

p65 – cytoplasmic protein

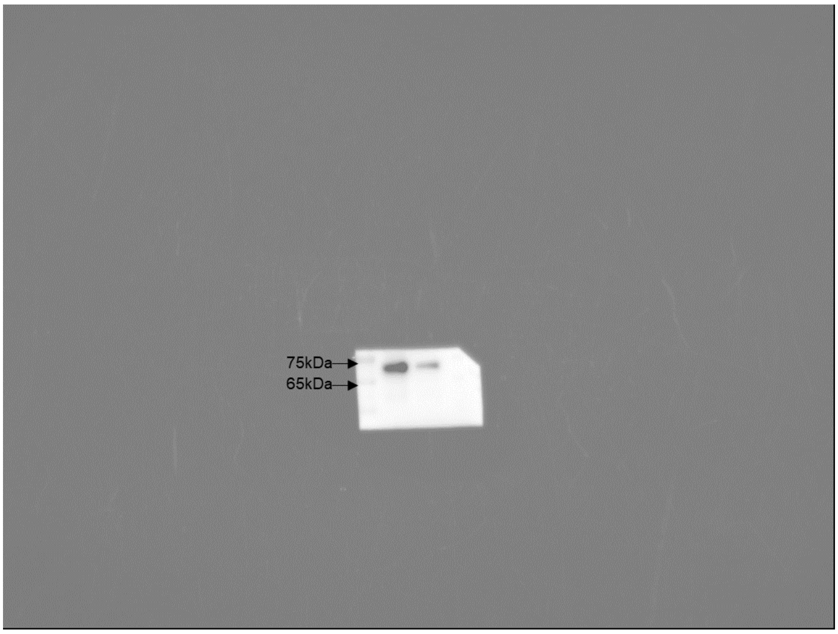

GAPDH – cytoplasmic protein

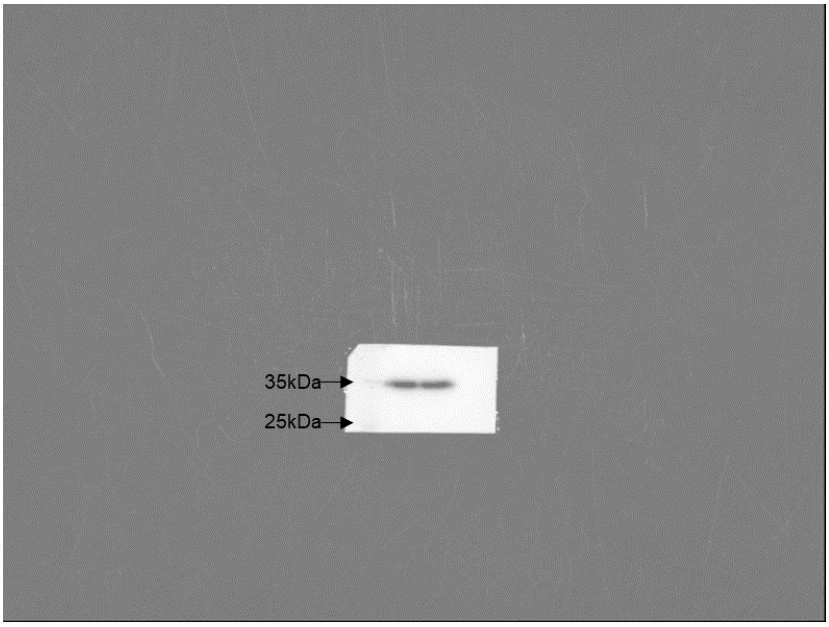

p65 - nuclear protein

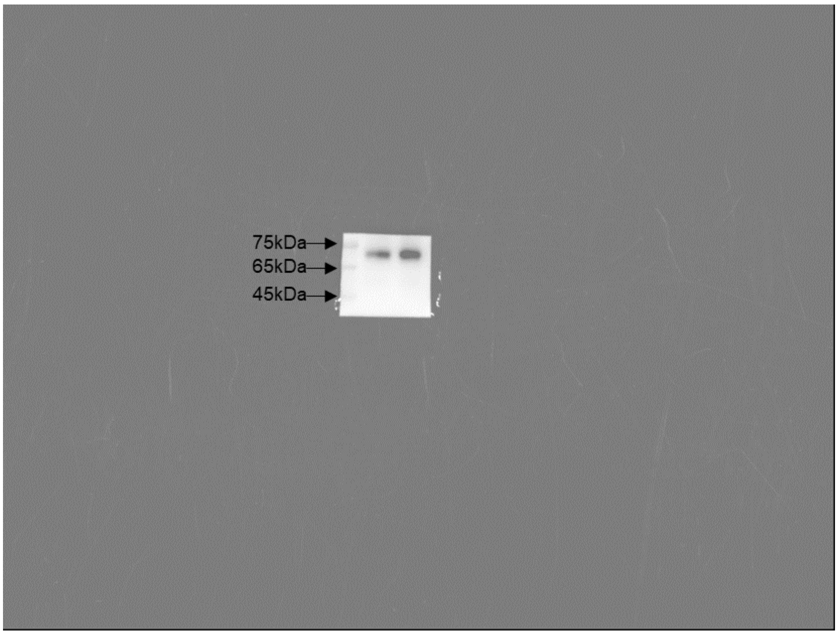

Snail - nuclear protein

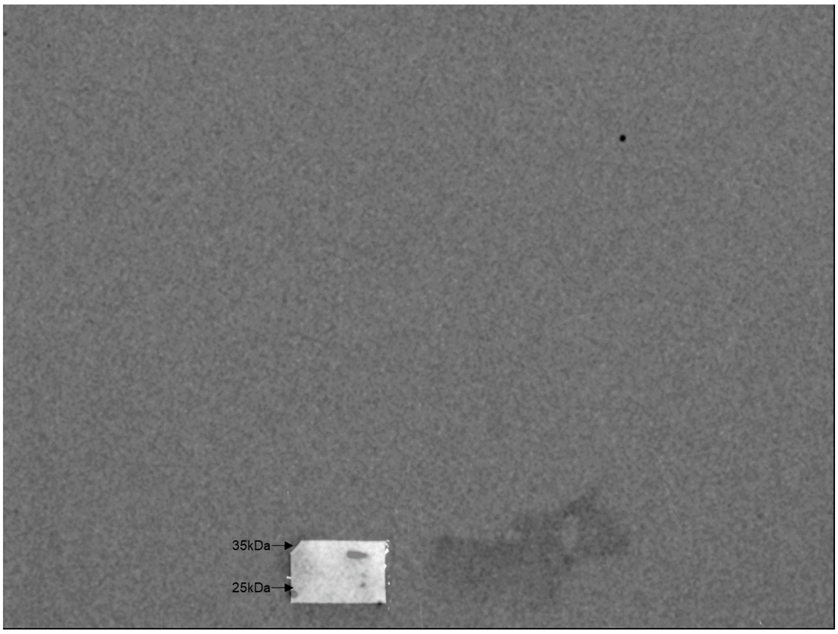

Figure S5 Original western blot figures for figure 3A.

p65 - cytoplasmic protein

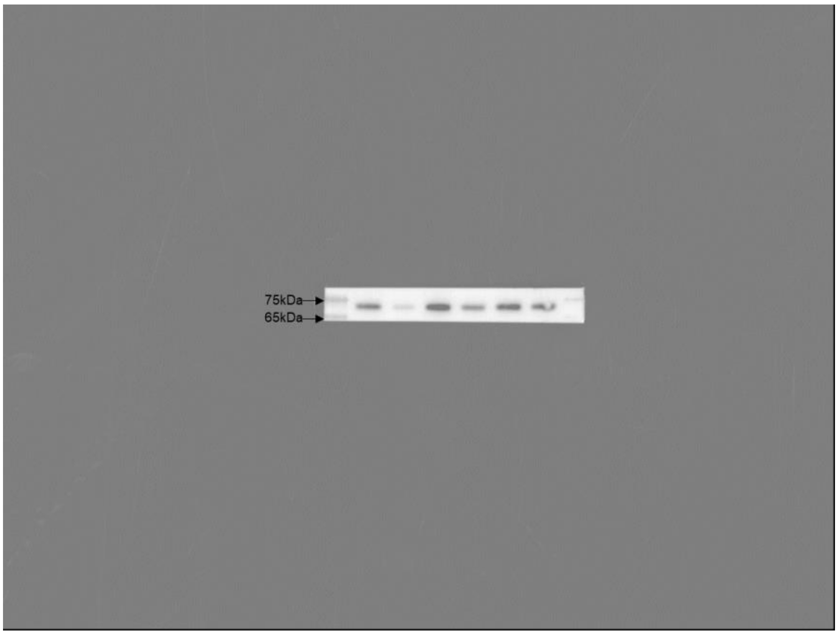

GAPDH - cytoplasmic protein

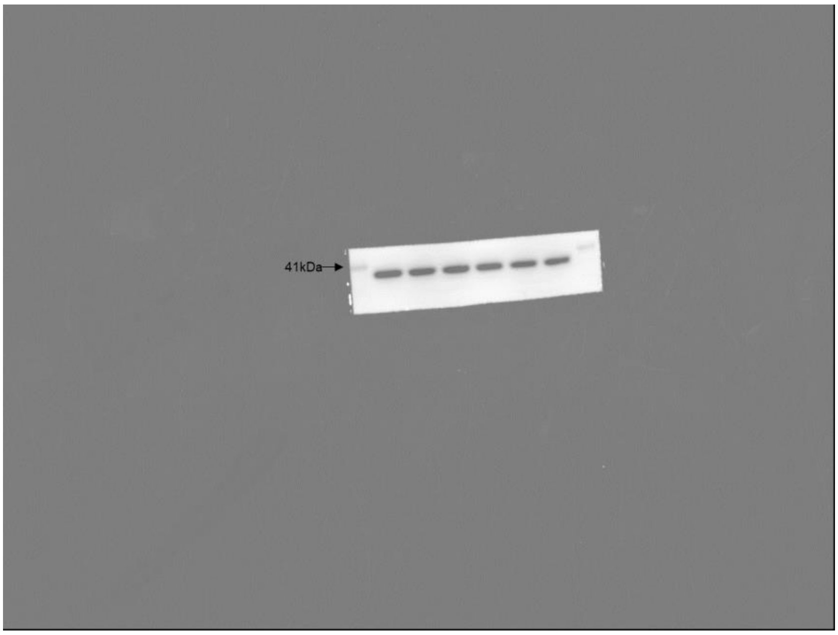

p65 – nuclear protein

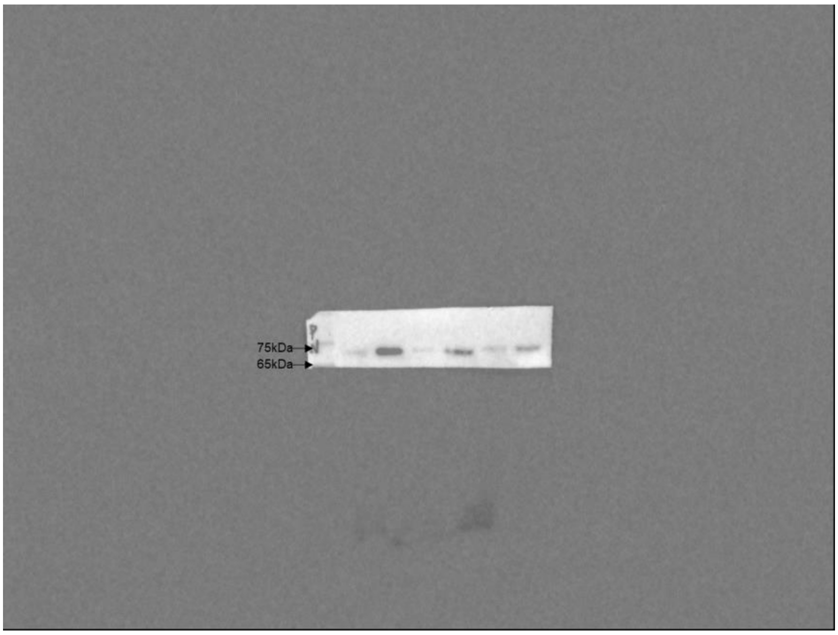

Snail – nuclear protein

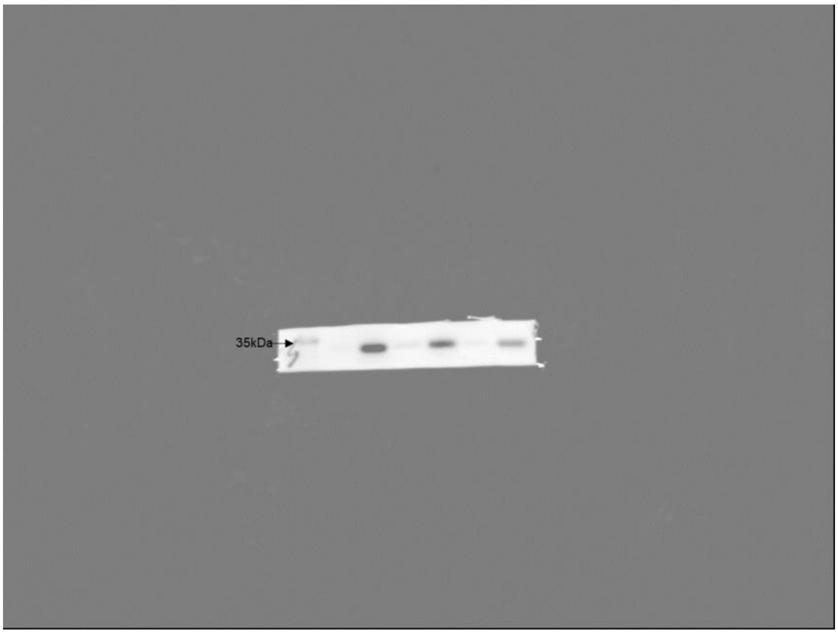

Histone H3 – nuclear protein

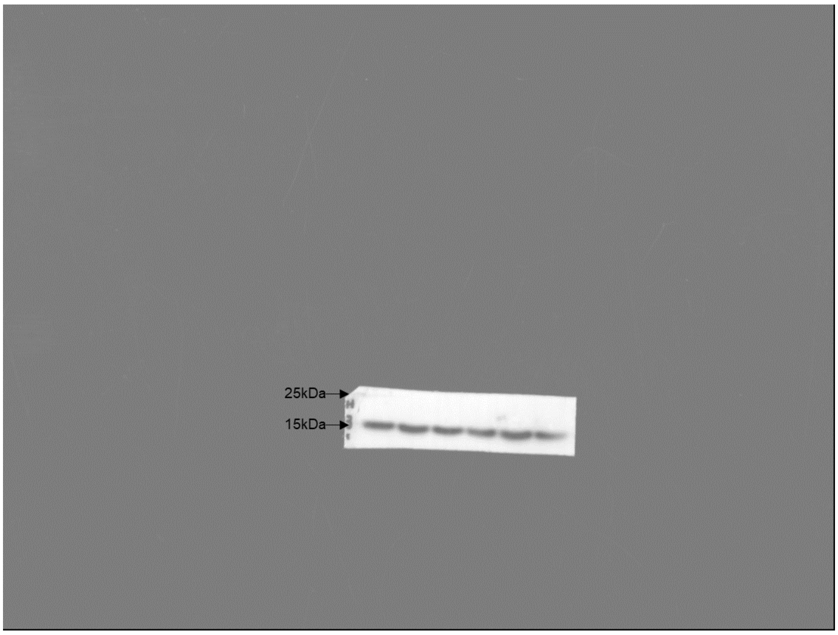

Figure S6 Original western blot figures for figure 3E.

E-cadherin

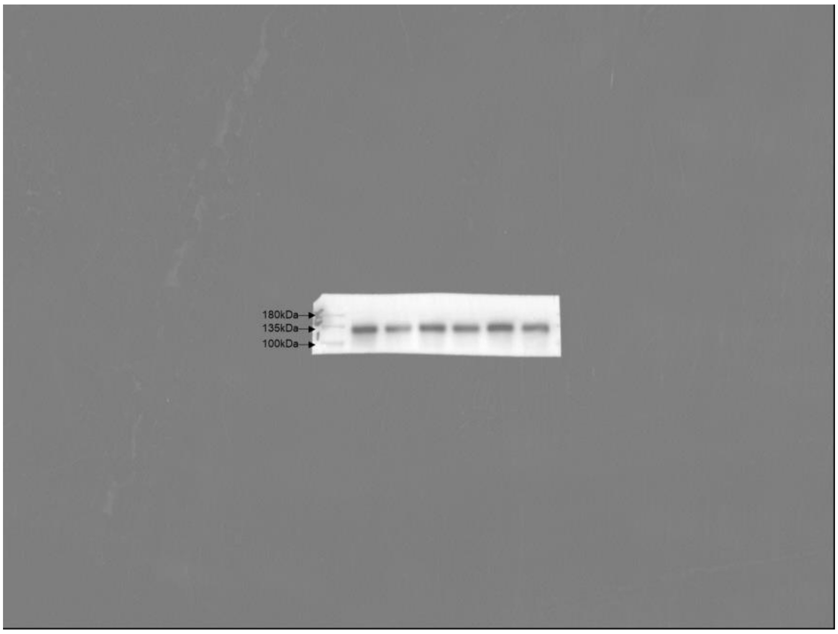

N-cadherin

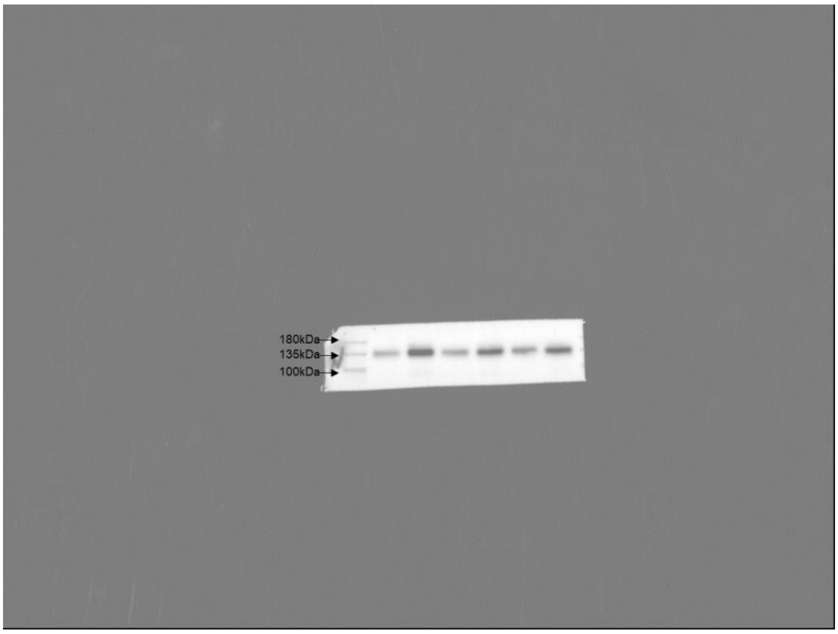

Vimentin

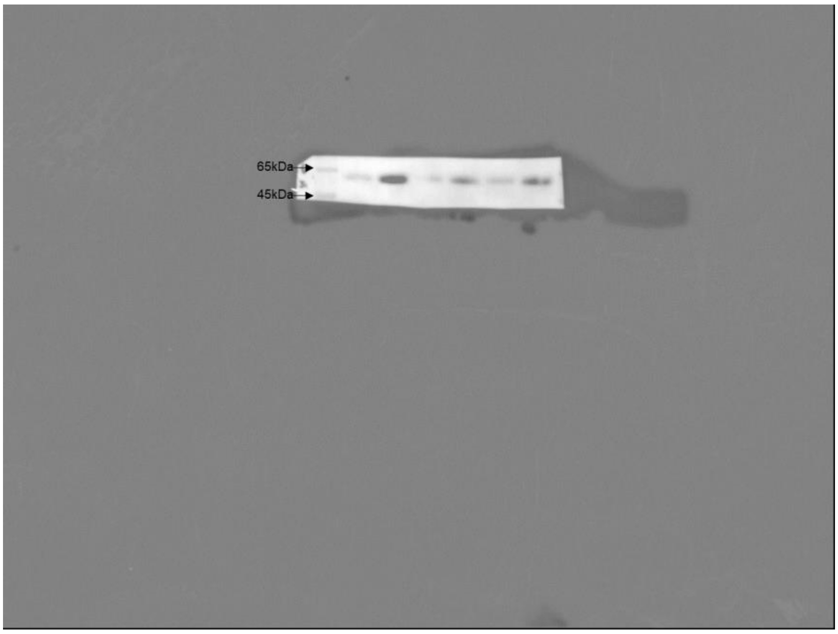

Collagen III

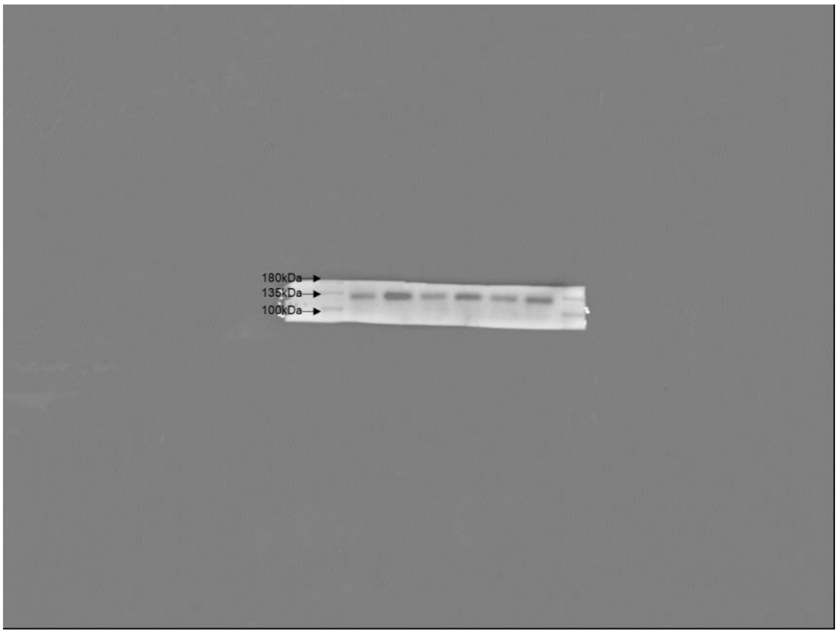

GAPDH

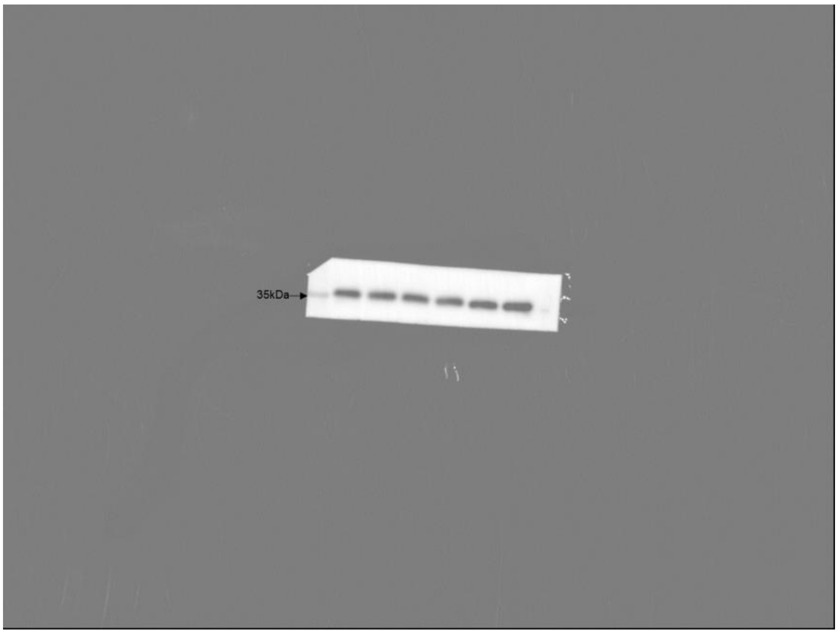

Figure S7 Original western blot figures for figure 3F.

pp65

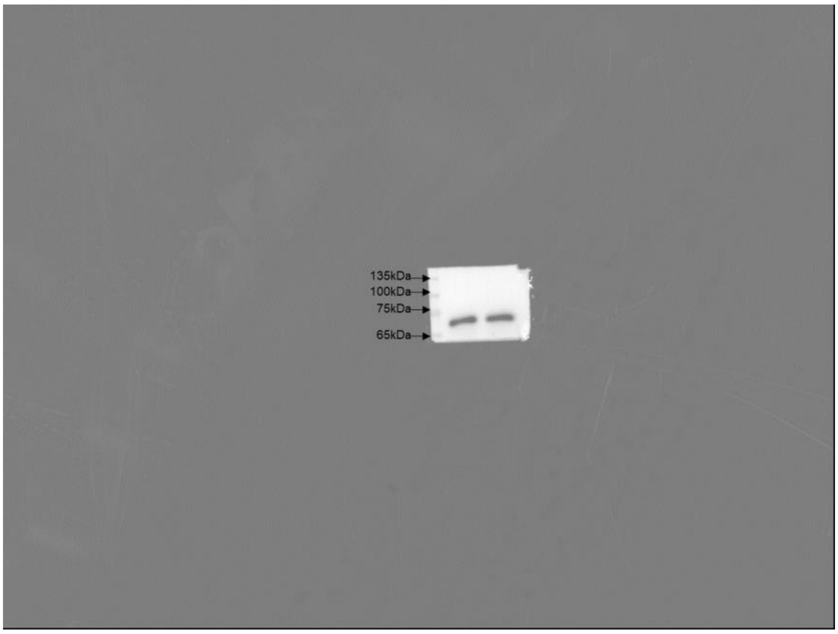

Snail

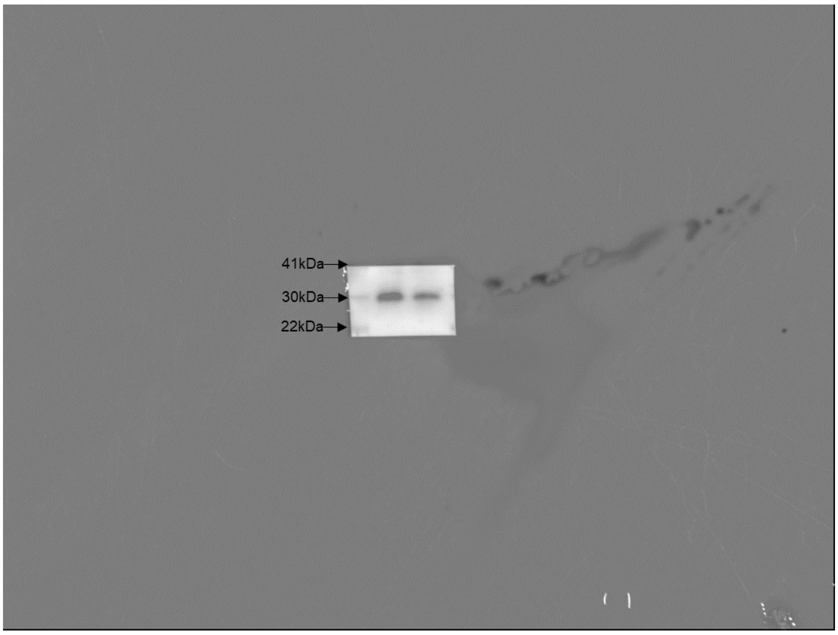

β-actin

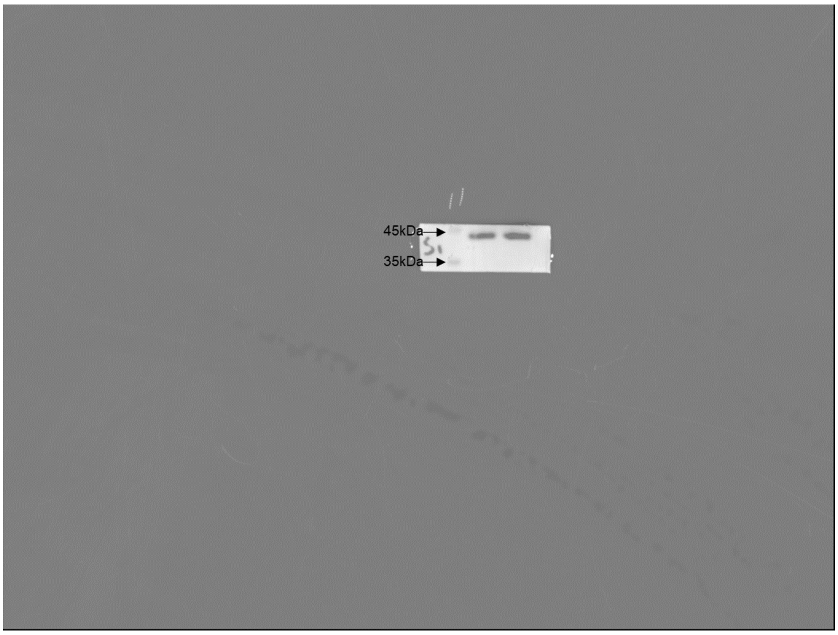

Figure S8 Original western blot figures for figure 4B.

E-cadherin

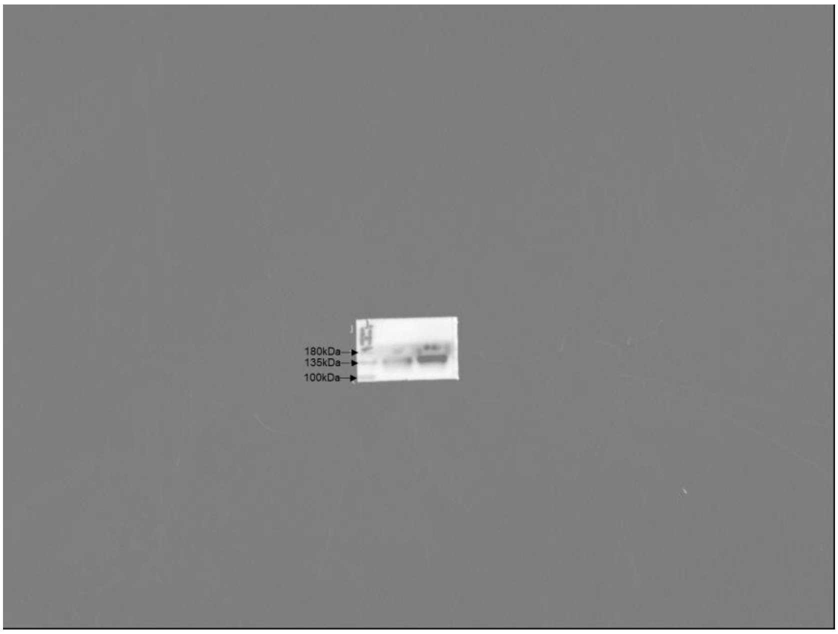

N-cadherin

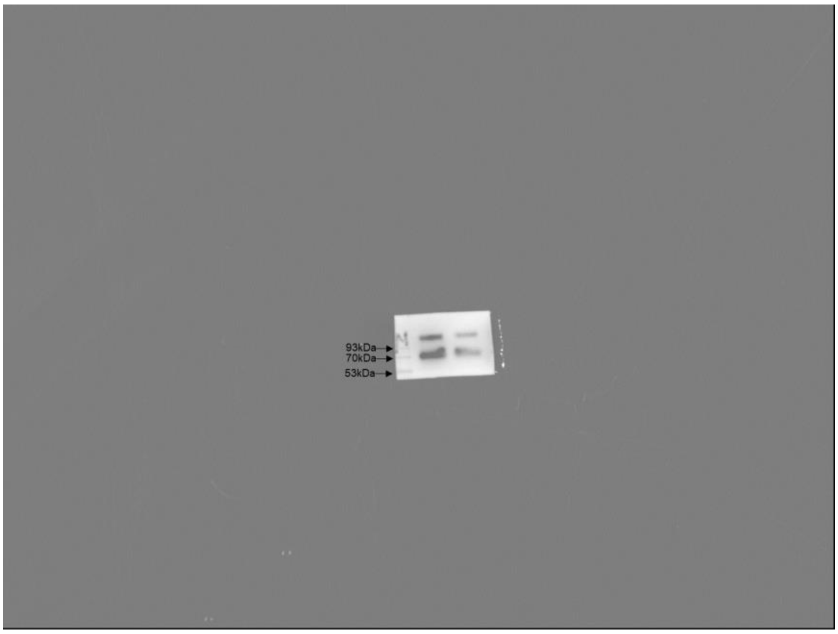

Collagen III

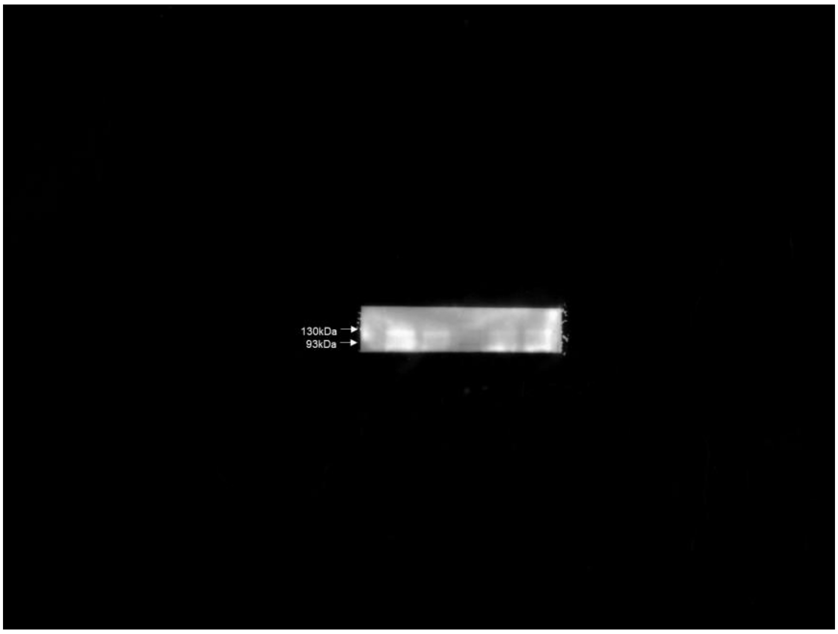

Vimentin

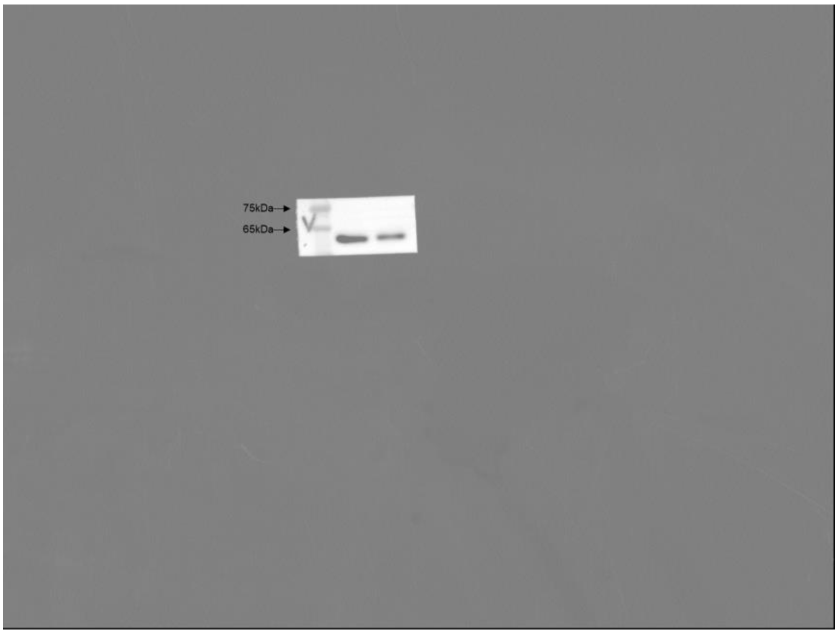

β-actin

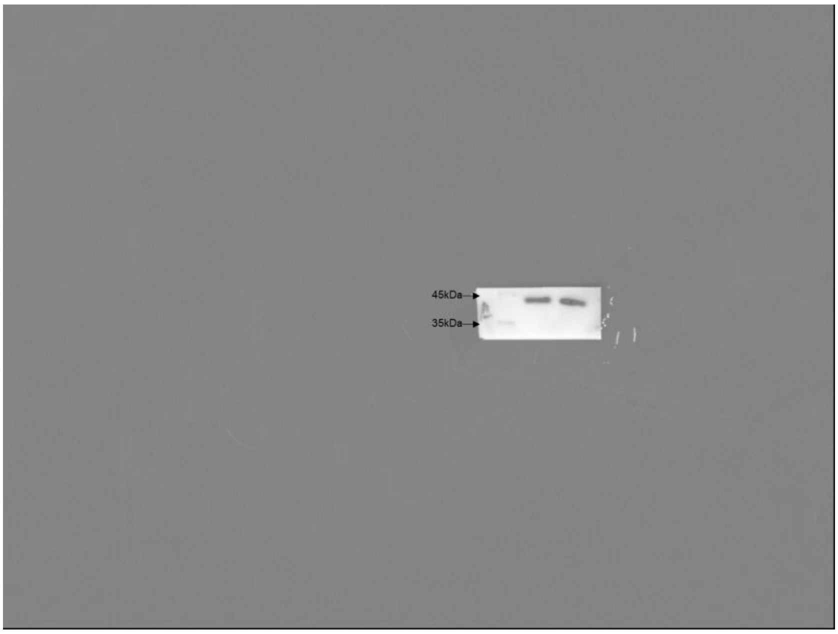

Figure S9 Original western blot figures for figure 4D.

pp65

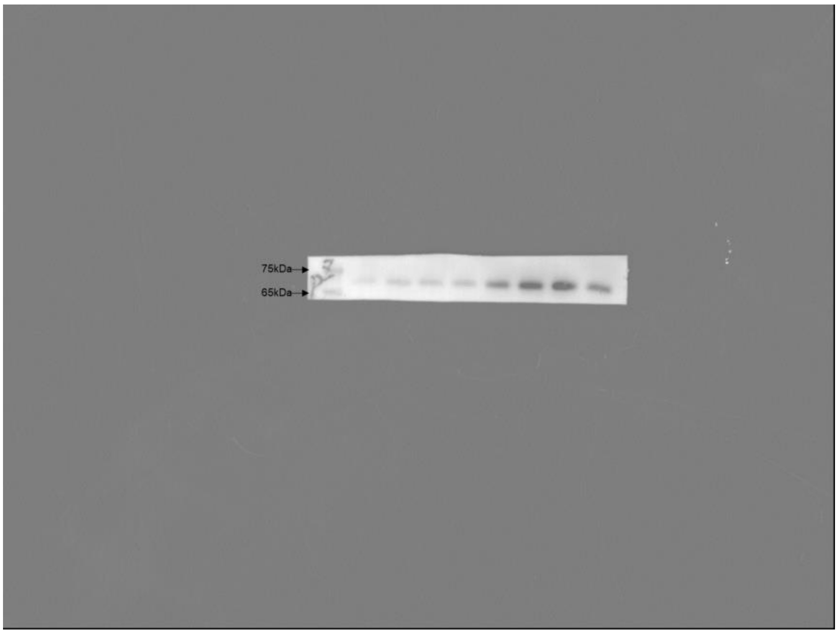

Snail

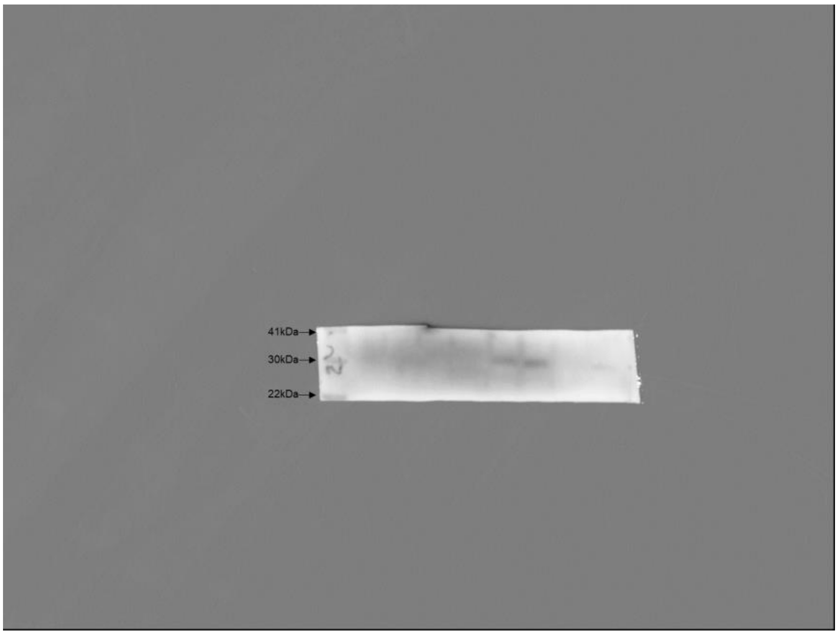

β-actin

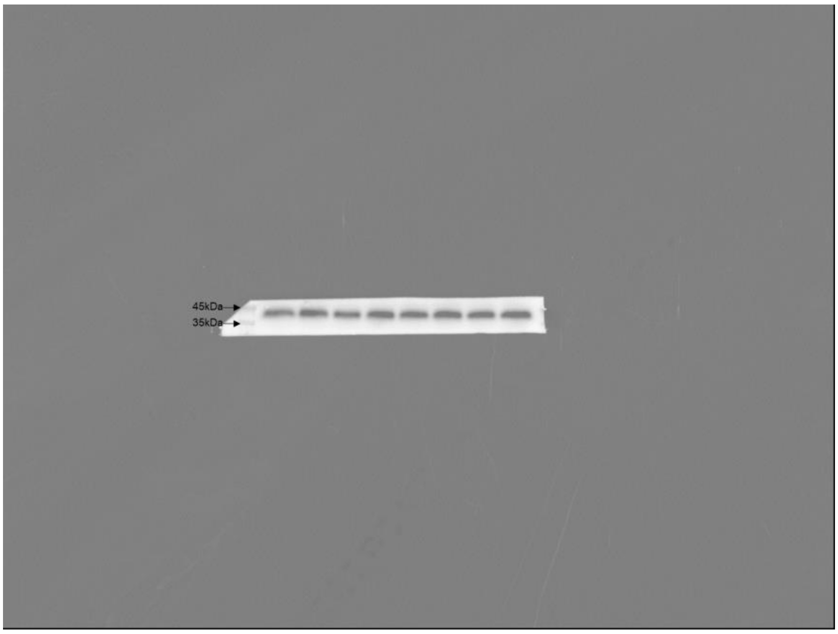

Figure S10 Original western blot figures for figure 5D.

β-casein

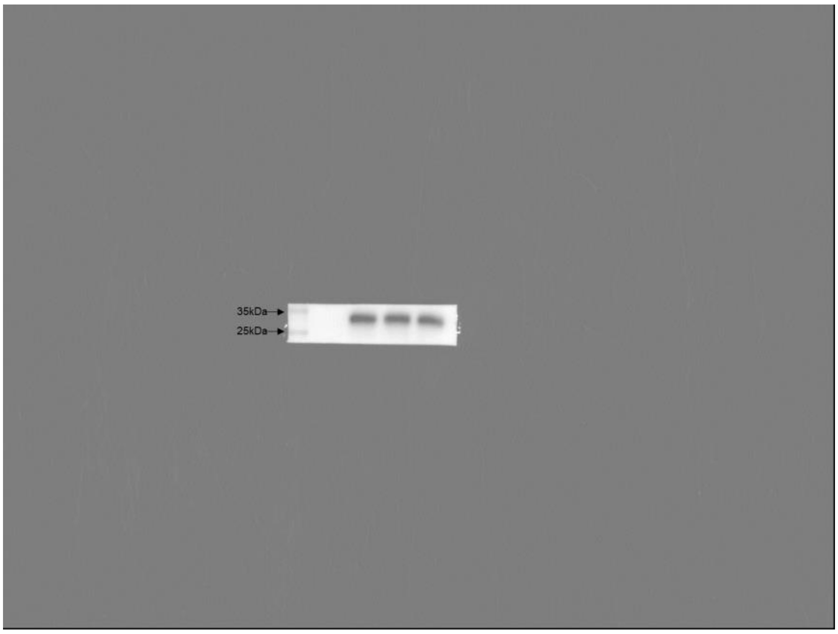

GAPDH

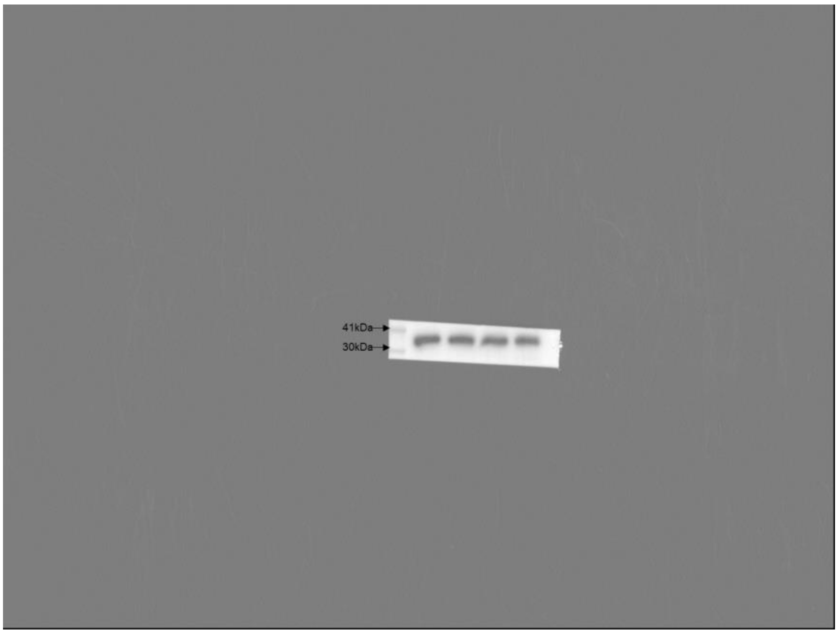

Figure S11 Original western blot figures for figure A2C.

pp65

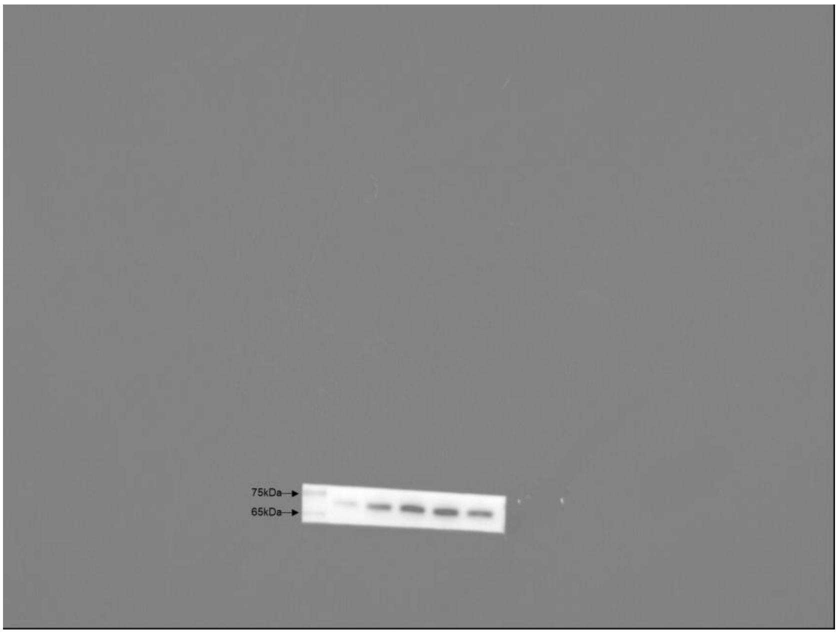

p65

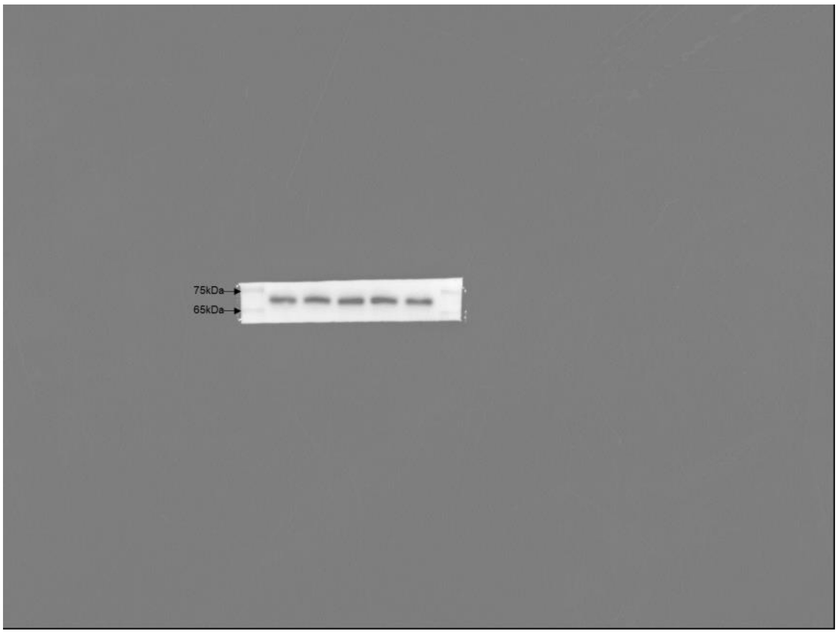

β-actin

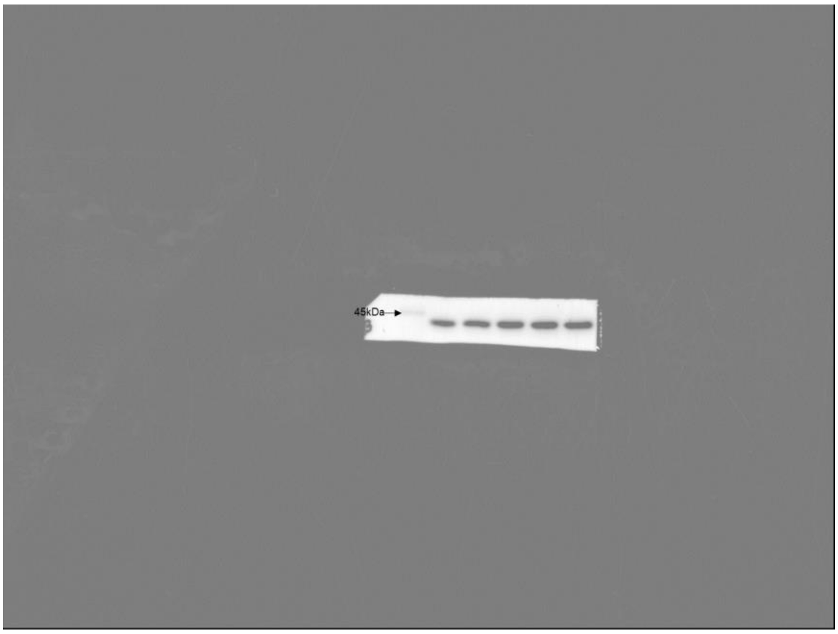

Figure S12 Original western blot figures for figure A3C.

pp65

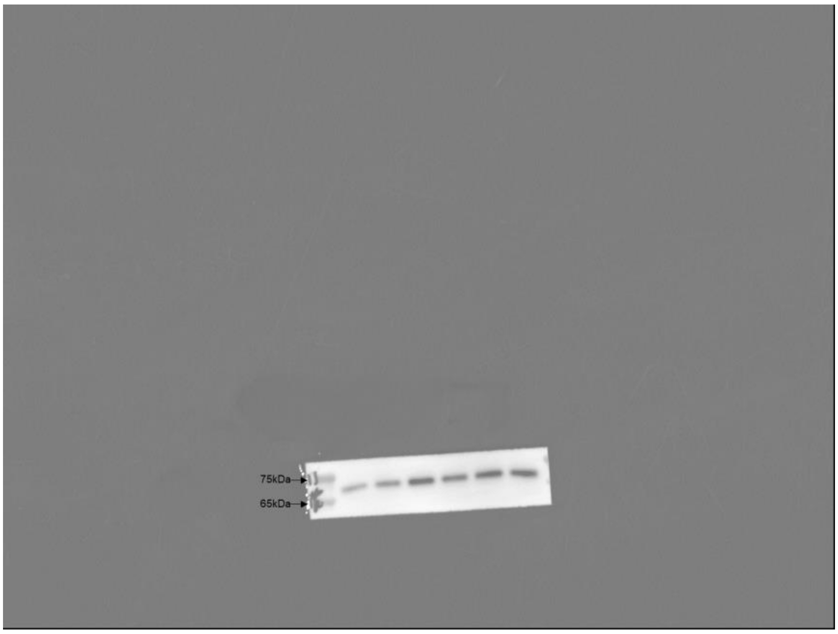

p65

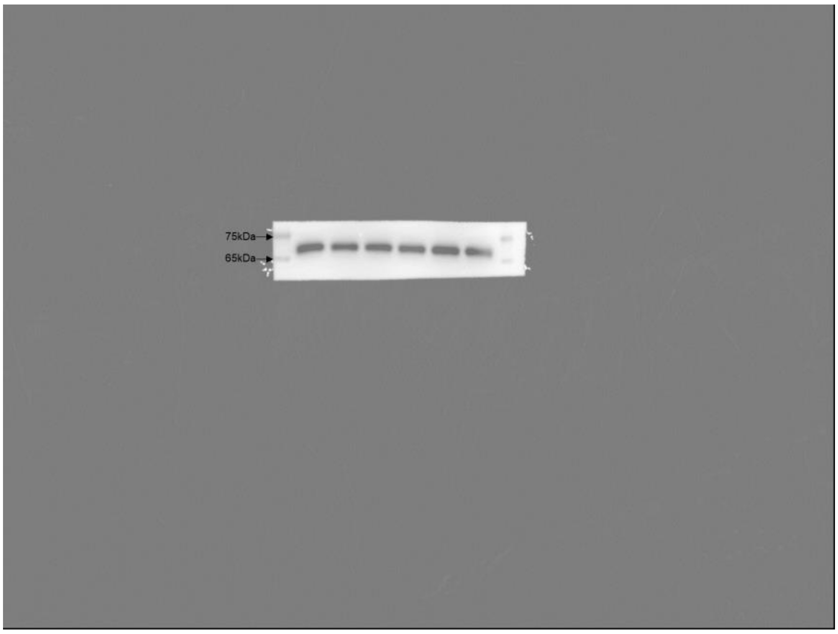

β-actin

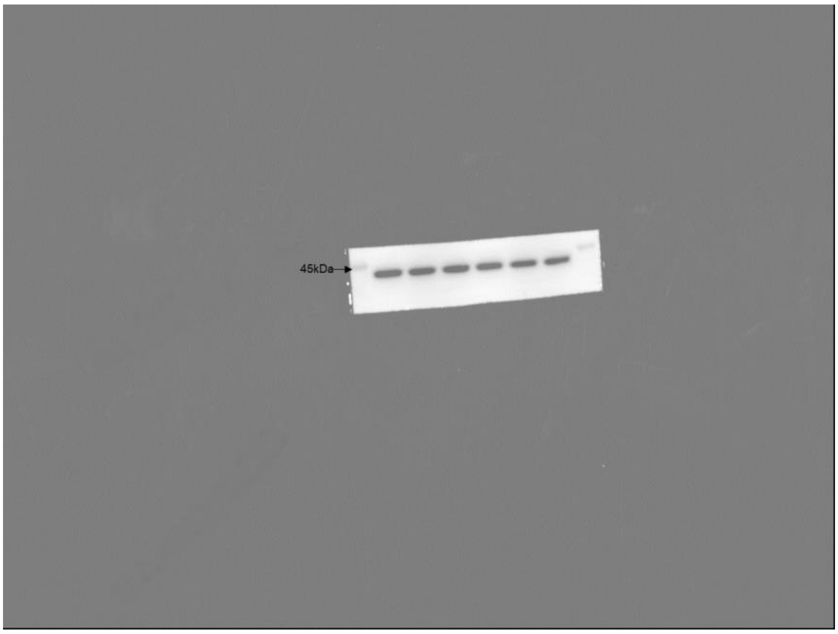

Figure S13 Original western blot figures for figure A3D.

α-SMA

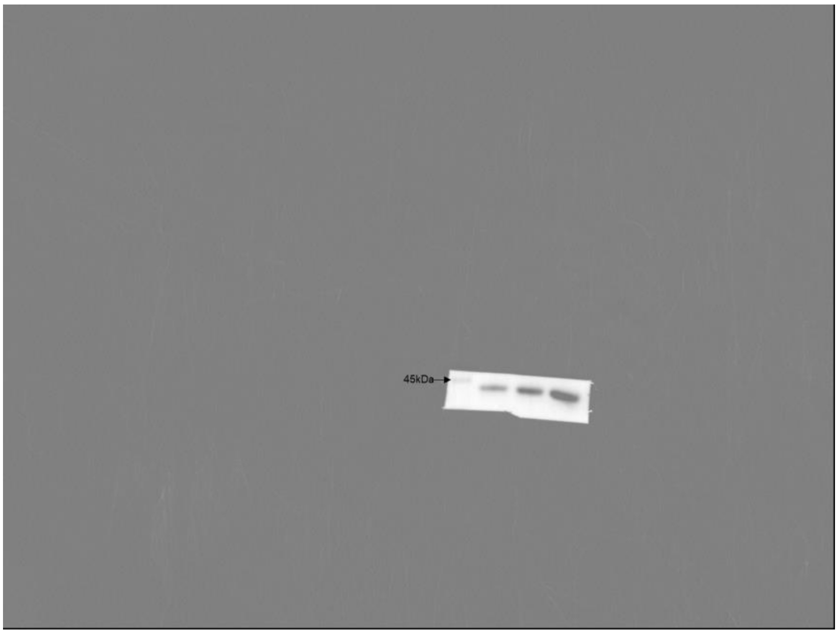

β-casein

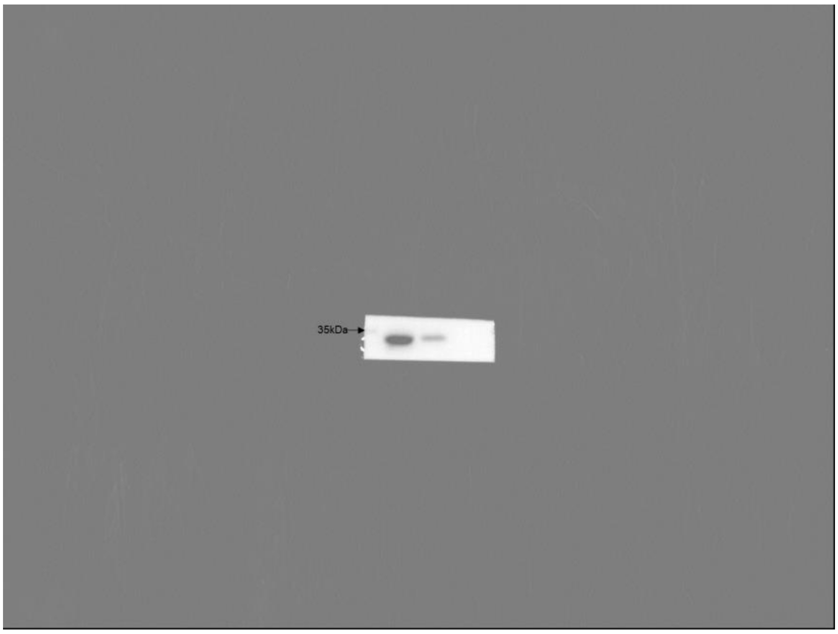

GAPDH

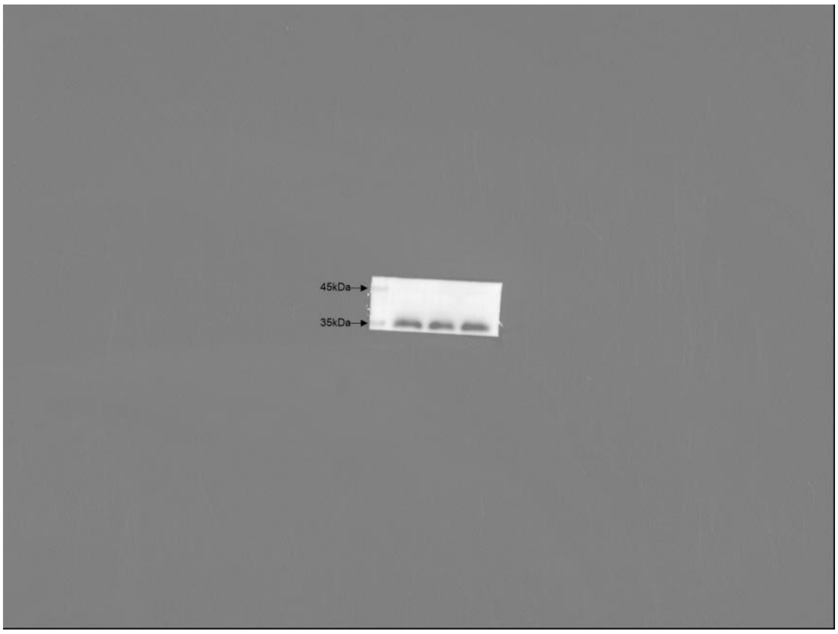

Figure S14 Original western blot figures for figure A4B.
